# 2448 The effect of allopurinol on pediatric patients undergoing maintenance chemotherapy for acute lymphoblastic leukemia or lymphoblastic lymphoma

**DOI:** 10.1017/cts.2018.187

**Published:** 2018-11-21

**Authors:** Mariam M. Bhuiyan, Gordon Cohen, Stacy Cooper

**Affiliations:** Johns Hopkins University School of Medicine

## Abstract

OBJECTIVES/SPECIFIC AIMS: This study aims to assess the safety, feasibility, clinical benefits and pharmacodynamics of adding allopurinol to standard maintenance therapy that includes 6-mecaptopurine (6-MP) in pediatric patients with acute lymphoblastic leukemia (ALL) or lymphoblastic lymphoma. Our goal is to investigate if allopurinol improves hepatotoxicity and GI toxicity, if it safely decreases acute neutrophil count (ANC), if it reduces the 6-MP dose required during chemotherapy, and if it works through our hypothesized mechanism by lowering the levels of the toxic metabolite, 6-methylmecaptopurine (6-MMP) and by raising the levels of the active metabolite, 6-thioguanine (6-TGN). METHODS/STUDY POPULATION: This is a single arm, nonblinded pilot study of patients under age 30 years who were being treated in the maintenance phase of therapy for ALL or lymphoblastic lymphoma, and had adverse effects such as high 6-MMP:6-TGN ratio, high ANC, and high liver enzymes. Patients enrolled were started with allopurinol in addition to ongoing oral chemotherapy. Data from beginning maintenance to end of chemotherapy was collected in the electronic medical record, EPIC for the 13 patients enrolled at Johns Hopkins, and data analysis was conducted using STATA and Excel. RESULTS/ANTICIPATED RESULTS: Initial data analysis reveals that the required dose of 6-MP after addition of allopurinol to the chemotherapy regimen was significantly lower compared with that before the addition of allopurinol in 11 out of the 12 patients assessed (*p*<0.05). Among the 10 patients that were assessed for 6MMP:6TG ratio, all had lower average 6MMP:6TGN ratios after allopurinol compared to before allopurinol; the percentage of weeks that goal 6MMP:6TGN ratio (<40) were maintained were statistically significant in 6 patients (*p*<0.05) and close to significance in 2 other patients (*p*=0.057). The percentage of weeks that patients maintained alanine aminotransferase levels below 120 was significantly greater after addition of allopurinol compared to before the addition of allopurinol in 9 out of 13 patients assessed, suggesting that allopurinol may be associated with reduced hepatotoxicity. Further data analysis is ongoing to assess the percentage of weeks that patients maintained goal total bilirubin, direct bilirubin, and ANC, as well as average number of admissions for infections and average number of therapy holds after allopurinol addition compared to before allopurinol addition. DISCUSSION/SIGNIFICANCE OF IMPACT: Allopurinol is associated with reduction in required 6-MP dose, decrease in the percentage of weeks that patients have hepatotoxicity, and reduction in the ratio of toxic metabolite to active anti-leukemic metabolite in several patients. We hope that the results of this study can be used for further research and for guiding clinical practice since there are no established guidelines in pediatric oncology regarding addressing side effects of oral chemotherapy using 6-MP. If allopurinol indeed is safe and effective, adding it to the standard chemotherapy regimen can lead to better tolerance and compliance to oral maintenance chemotherapy, and hopefully improved outcomes for children with ALL and lymphoblastic leukemia.

